# Effects of disrupting the polyketide synthase gene *WdPKS1 *in *Wangiella *[*Exophiala*] *dermatitidis *on melanin production and resistance to killing by antifungal compounds, enzymatic degradation, and extremes in temperature

**DOI:** 10.1186/1471-2180-6-55

**Published:** 2006-06-19

**Authors:** William F Paolo, Ekaterina Dadachova, Piyali Mandal, Arturo Casadevall, Paul J Szaniszlo, Joshua D Nosanchuk

**Affiliations:** 1 Department of Medicine, Albert Einstein College of Medicine, Bronx, NY, USA; 2 Department of Microbiology and Immunology, Albert Einstein College of Medicine, Bronx, NY, USA; 3 Department of Nuclear Medicine, Albert Einstein College of Medicine, Bronx, NY, USA; 4 Department of Microbiology, All India Institute of Medical Sciences and Division of Infectious Diseases, New Delhi, India; 5 Section of Molecular Genetics and Microbiology, The University of Texas at Austin, Austin, TX, USA; 6 Department of Medicine, New York University Medical Center, NY, USA

## Abstract

**Background:**

*Wangiella dermatitidis *is a human pathogenic fungus that is an etiologic agent of phaeohyphomycosis. *W. dermatitidis *produces a black pigment that has been identified as a dihydroxynaphthalene melanin and the production of this pigment is associated with its virulence. Cell wall pigmentation in *W. dermatitidis *depends on the *WdPKS1 *gene, which encodes a polyketide synthase required for generating the key precursor for dihydroxynaphthalene melanin biosynthesis.

**Results:**

We analyzed the effects of disrupting *WdPKS1 *on dihydroxynaphthalene melanin production and resistance to antifungal compounds. Transmission electron microscopy revealed that *wdpks1*Δ*-1 *yeast had thinner cell walls that lacked an electron-opaque layer compared to wild-type cells. However, digestion of the *wdpks1*Δ*-1 *yeast revealed small black particles that were consistent with a melanin-like compound, because they were acid-resistant, reacted with melanin-binding antibody, and demonstrated a free radical signature by electron spin resonance analysis. Despite lacking the *WdPKS1 *gene, the mutant yeast were capable of catalyzing the formation of melanin from L-3,4-dihyroxyphenylalanine. The *wdpks1*Δ*-1 *cells were significantly more susceptible to killing by voriconazole, amphotericin B, NP-1 [a microbicidal peptide], heat and cold, and lysing enzymes than the heavily melanized parental or complemented strains.

**Conclusion:**

In summary, *W. dermatitidis *makes *WdPKS*-dependent and -independent melanins, and the *WdPKS1-*dependent deposition of melanin in the cell wall confers protection against antifungal agents and environmental stresses. The biological role of the *WdPKS*-independent melanin remains unclear.

## Background

*Wangiella *[*Exophiala*] *dermatitidis *is a polymorphic, dematiaceous [darkly pigmented] fungal pathogen of humans that exists predominantly as a yeast form in vitro, but produces various morphological structures, such as pleomorphic yeast, pseudohyphae, true hyphae, and sclerotic bodies in tissues [1]. The dark pigmentation of *W. dermatitidis *is considered to be due to deposition of 1,8-dihydroxynaphthalene [1, 8-DHN] melanin in the cell wall of the organism [2-4]. Melanins are multifunctional polymers that are found in species from all biological kingdoms [5]. Whereas human melanin synthesis is solely catalyzed by tyrosinase, microbes primarily synthesize melanin via phenoloxidases [such as tyrosinases, laccases or catacholases] and/or a polyketide synthase pathway. Pigments derived from acetate via the polyketide synthase pathway are generally black or brown and are generally referred to as dihydroxynaphthalene [DHN] melanins. Eumelanins, also called DOPA melanins, are characteristically black or brown and are formed by the oxidative polymerization of phenolic and/or indolic compounds catalyzed by phenoloxidases. Nonetheless, both types of melanins are negatively charged, hydrophobic pigments of high molecular weight [6-8]. The production of melanin is associated with *W. dermatitidis *virulence wherein the absence of the pigment in the cell wall results in increased killing of the fungus by host cells and decreased disease severity [2,9-13]. Melanins are important for virulence of some other human pathogenic fungi, such as *Cryptococcus neoformans*, *Aspergillus *species, and *Sporothrix schenckii *[reviewed in [14]]. Melanins have also been implicated in the virulence of several fungal plant pathogens [6,15].

Diverse compounds have been shown to interact with melanins, including antifungal drugs [16-18]. Traditionally, amphotericin B has been the major antifungal drug used in the treatment of *W. dermatitidis *infections [19]. However, recently the in vitro efficacy of amphotericin B was shown to be reduced by melanization in *C. neoformans *[16-18], *Histoplasma capsulatum *[16], and *Blastomyces dermatitidis *[20]. Although fluconazole and caspofungin are not effective in vivo against *W. dermatitidis*, melanization can protect *C. neoformans *and *H. capsulatum *against these antifungal drugs in vitro [16,18]. Additionally, the activity of microbicidal peptides has been shown to be reduced in melanized *C. neoformans *yeast cells [21].

The gene *WdPKS1*, which has been cloned, sequenced and disrupted previously, encodes a polyketide synthase [WdPks1p] in *W. dermatitidis *that is considered to be the major enzyme controlling melanin production in this fungus [2]. Compared to their wild-type parent, *wdpks1*Δ mutants are more susceptible to neutrophil killing and less virulent in a mouse infection model, but the gross cellular morphology of the *wdpks1*Δ cells is not affected by the loss of WdPks1p. In this study, we further characterize the structural differences and production of melanins among the *wdpks1*Δ-*1 *mutant, the *wdpks1*Δ-*1 *mutant complemented with the *WdPKS1 *wild-type gene, and their intact *W. dermatitidis *parental strain. We report the surprising finding that *W. dermatitidis *produces melanin independent of *WdPKS1 *activity. Additionally, we evaluate the role of *W. dermatitidis *cell wall melanin in protection against antifungal compounds, heat and cold, and osmotic stress.

## Results

### *Wangiella *melanization and isolation of melanin-like particle

**Figure 1:**
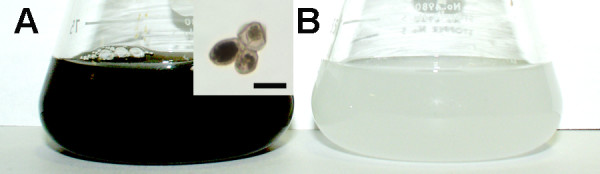
*W. dermatitidis *strain *wdpks1*Δ-*1 *after growth for 10 days in a defined chemical medium with [A] and without [B] L-DOPA. The inset with light microscopy in [A] depicts particles isolated from the culture after serial treatment with enzymes, denaturant, and hot acid; scale bar, 5 μm.

**Figure 2:**
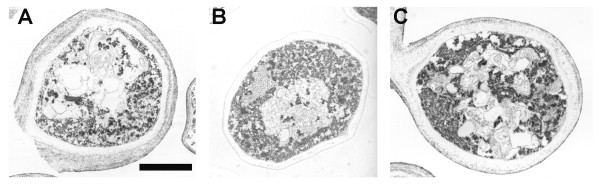
Representative transmission electron micrographs of *W. dermatitidis *strains A] 8656, B] *wdpks1*Δ-*1*, and C] *wdpks1*Δ-*1*-501 grown for 7 days in YPD medium at 37°C. Scale bar, 2 μm.

Yeast cells of wild type strain 8656 and the *wdpks1*Δ*-1*-501 complemented strain became black after 3 days of growth at 37°C in YPD broth. In contrast, the *wdpks1*Δ*-1 *yeast similarly cultured did not turn black. Instead, after 5 to 7 days of growth in YPD, the *wdpks1*Δ*-1 *yeast began to develop a light brown coloration. However, when grown in minimal medium with L-3,4-dihyroxyphenylalanine [L-DOPA], the *wdpks1*Δ*-1 *yeast were grey after 3 to 4 days and black after 7 days whereas they were white in the absence of L-DOPA [Figure 1]. Transmission electron microscopy revealed that the yeast cell walls of strains 8656 and *wdpks1*Δ*-1*-501 grown for 7 days in YPD were significantly thicker than those of the *wdpks1*Δ*-1 *strain [Figure 2]. Measurement of twenty non-serial cell sections of each strain showed that the wall thicknesses for strains 8656, *wdpks1*Δ*-1*, and *wdpks1*Δ*-1*-501 were 227 ± 13 nm, 141 ± 13, and 229 ± 19 nm, respectively [p < 0.001, for strain 8656 compared to *wdpks1*Δ*-1 *by Student's *t *test]. In addition to being thicker, layers of the cell walls of strains 8656 and *wdpks1*Δ*-1*-501 were dramatically more electron dense than in the melanin deficient strain.

**Figure 3:**
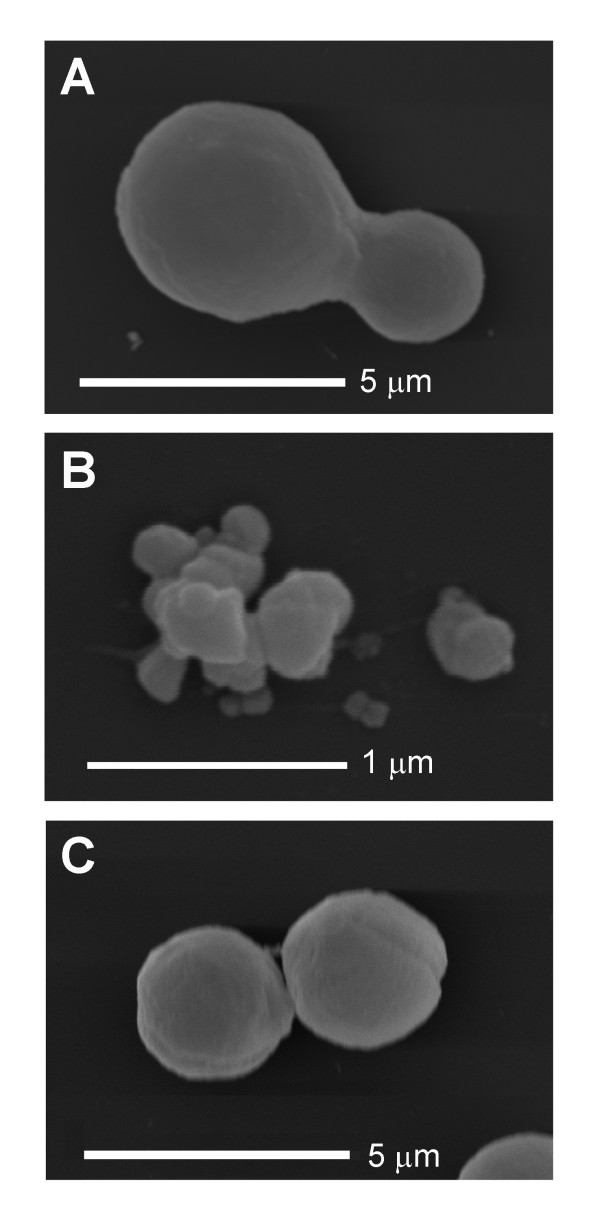
Representative scanning electron microscopy of particles collected after treatment with enzymes, denaturant, and hot acid from *W. dermatitidis *strains A] 8656, B] *wdpks1*Δ-*1*, and C] *wdpks1*Δ-*1*-501 grown for 10 days in YPD medium at 37°C.

Treatment of cells from 10-day cultures of the black strains 8656 and *wdpks1*Δ*-1*-501 grown in YPD with enzymes, denaturant and hot acid produced cell residues that were similar in size and shape to unextracted yeast [Figure 3A and 3C]. In contrast, treatment of the *wdpks1*Δ*-1 *cells grown identically yielded black particles that were significantly smaller than the cells from which they were derived [Figure 3B]. Instead of the yeast-like forms seen with the black strains, the particles derived from the melanin-deficient strain varied greatly in size and were globular and irregular in shape. However, when grown in defined chemical medium with L-DOPA, treatment of *wdpks1*Δ*-1 *yeast resulted in particles similar in shape to their propagules that had grayish outer spherical layer with dense black pigment within [Figure 1A, inset]. The *wdpks1*Δ*-1 *yeast are completely solubilized when grown in the chemical medium without L-DOPA [data not shown]. Melanins from cells grown in YPD for 10 days were subjected to elemental analysis. Quantitative analysis revealed that melanin comprised 16.1 ± 0.4% of the dry mass of wild type strain 8656 yeast cells. The weight of *wdpks1*Δ*-1*-501 complemented strain similarly consisted of 15.1 ± 0.2% melanin, whereas the pigment constituted 4.6 ± 0.1% of the *wdpks1*Δ*-1 *yeasts' dry mass.

### Immunofluorescence analysis of melanin-like particles

**Figure 4:**
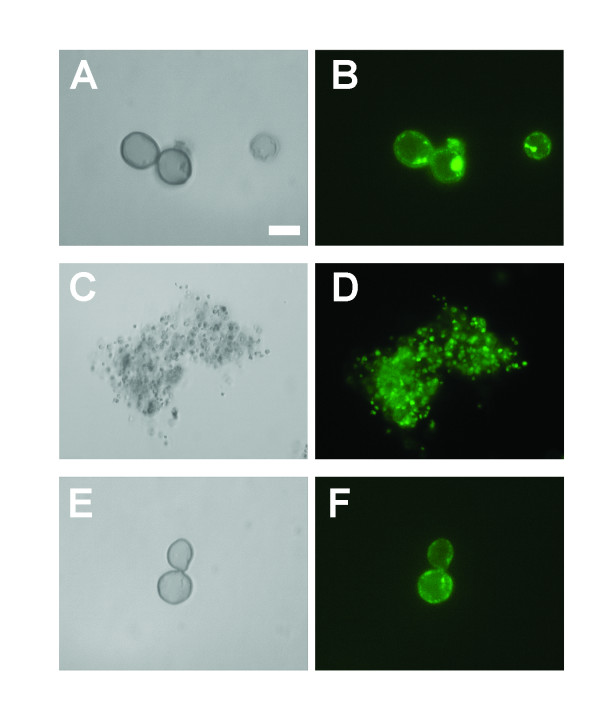
Corresponding light and immunofluorescence microscopy images of particles collected after treatment with enzymes, denaturant, and hot acid from *W. dermatitidis *strains [A, B] 8656, [C, D] *wdpks1*Δ-*1*, and [E, F] *wdpks1*Δ-*1*-501 grown for 10 days in YPD medium at 37°C. The particles were reacted with the melanin-binding mAb 6D2. Scale bar, 5 μm.

The melanin-like particles derived from the three strains were analyzed for their reactivity with a mAb specific for melanin by immunofluorescence. As expected, the particles from strains 8656 and *wdpks1*Δ*-1*-501 reacted with the mAb [Figure 4A,B and 4E,F, respectively]. The smaller particles isolated from *wdpks1*Δ*-1 *cells also reacted with the mAb [Figure 4C,D] suggesting that the particles are comprised of melanin-like compounds. No reactivity occurred when the control mAb or secondary antibody alone were used [data not shown].

**Figure 5:**
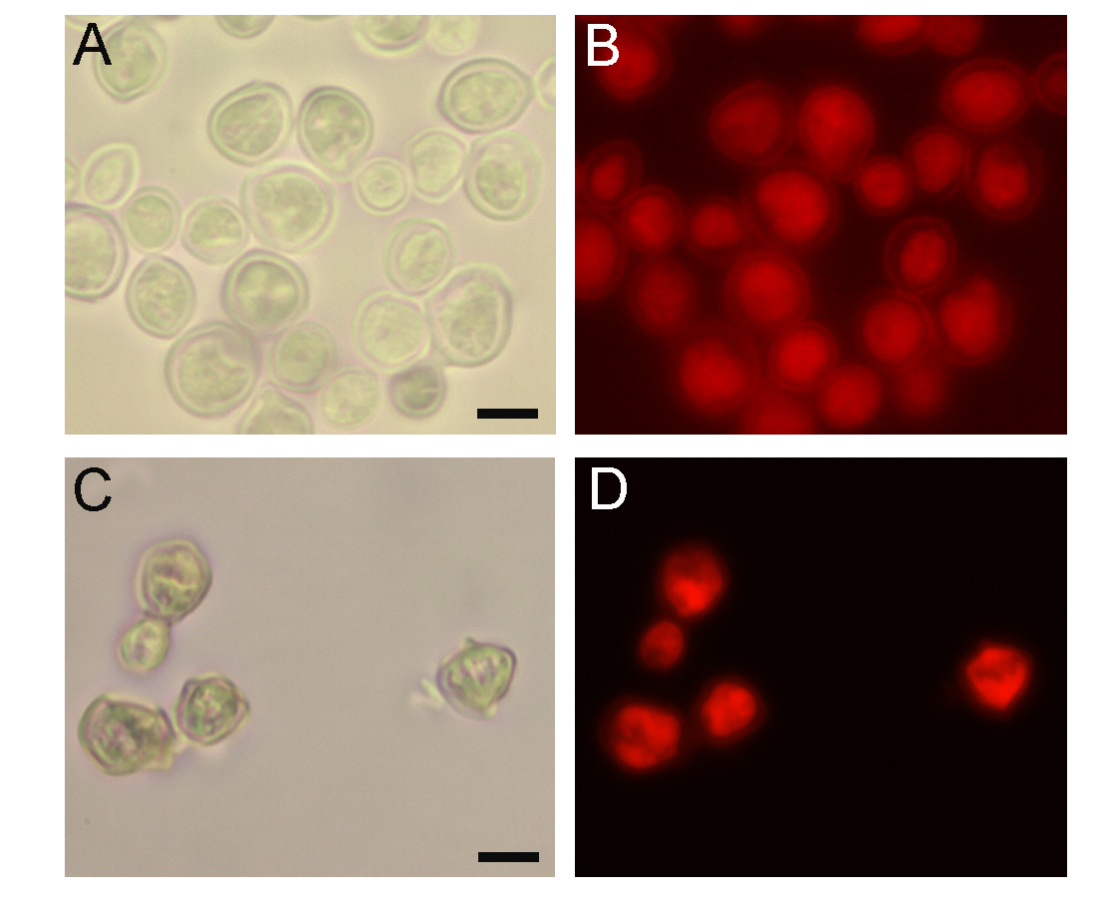
Corresponding light and immunofluorescence microscopy images of *W. dermatitidis *yeast cells of strain 8656 [A, B] and *wdpks1*Δ-*1 *[C, D] grown for 15 days in YPD medium at 37°C that were exposed to liquid nitrogen for 10 minutes prior to incubation with the melanin-binding mAb 6D2. Scale bar, 5 μm.

Imaging of the 15-day-old cells disrupted with liquid nitrogen revealed that all cells reacted with the melanin-binding antibody [Figure 5]. The disrupted wild-type cells demonstrated reactivity with the melanin-binding mAb both at the cell wall and within the cytoplasm [Figure 5B], whereas the cytoplasm of the melanin-deficient *wdpks1*Δ*-1 *cells were labeled [Figure 5D]. Additionally, light microscopy shows that the wild-type cells were significantly more resistant to damage induced by freezing in liquid nitrogen than the melanin-deficient mutant. In fact, the melanin-lacking cells collapsed after liquid nitrogen treatment, whereas those having melanin did not. As expected, cells from the *WdPKS1*-complemented strain *wdpks1*Δ*-1*-501 appeared similar to the wild-type yeast [data not shown]. No reactivity occurred when the control mAb or secondary alone were used [data not shown].

### Electron spin resonance [ESR] spectroscopy

**Figure 6:**
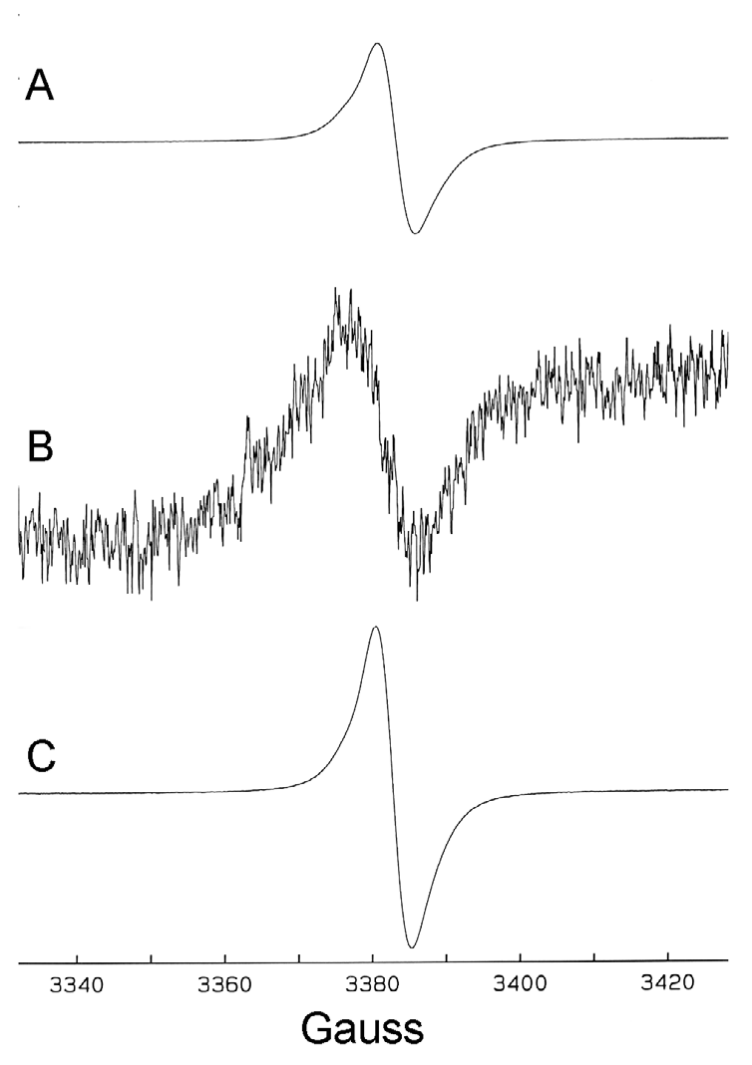
Electron spin resonance spectroscopy on particles collected after treatment with enzymes, denaturant, and hot acid from *W. dermatitidis *strains A] 8656, B] *wdpks1*Δ-*1*, and C] *wdpks1*Δ-*1*-501 grown for 10 days in YPD medium at 37°C. The spectra *wdpks1*Δ-*1 *is noisy because of the higher amplitude setting used to detect the weaker signal generated by the isolated melanin-like material.

An ESR spectroscopy spectrum generated by the black particles collected from strain 8656 *W. dermatitidis *was consistent with that of a melanin pigment [Figure 6A] [22], and was nearly identical to the signals generated with particles from *C. neoformans *[23], *P. brasiliensis *[24], *H. capsulatum *[25], *S. schenckii *[26], *Pneumocystis spp*. [27], and *Scytalidium dimidiatum *[28]. Although of lower amplitude, the signal generated with the particles isolated from the *wdpks1*Δ*-1 *yeast cells [Figure 6B] was similar to that seen with the particles purified from the pigmented 8656 and *wdpks1*Δ*-1*-501 strains [Figure 6A and 6C]. The lower amplitude from the melanin isolated from the *wdpks1*Δ*-1 *cells probably reflects the fact that there was a smaller quantity of particles available for ESR testing or structural differences of the material.

### Elemental analysis of melanins

Elemental quantitative analyses were performed to determine the relative elemental composition of the melanins. A significant percentage of nitrogen was detected in all isolates. The C:N ratios of the particles exposed to enzymes, denaturants, and acid were calculated and showed that the ratio for the wild type and complemented isolates were 18.6:1 and 19:1, respectively. In contrast, the ratio from the pigment isolated from the mutant *wdpks1*Δ*-1 *yeast cells was 26.2:1.

### Quantitative phenoloxidase assay and PAGE analysis for phenoloxidase-like activity

**Figure 7:**
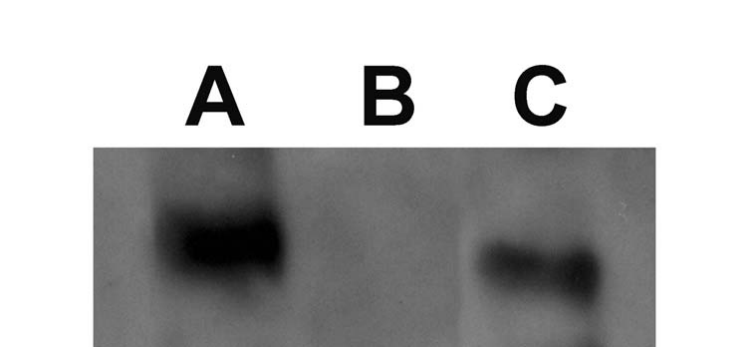
Nonreducing sodium dodecyl sulfate-PAGE gel of CYE of *W. dermatitidis *developed with L-DOPA. Synthesis of a black pigment consistent with melanin occurs in situ on the gel. Lane A, commercial laccase; lane B, 200 μg of *W. dermatitidis *strain *wdpks1*Δ-*1 *CYE of treated with KCN; lane C, 200 μg of *W. dermatitidis *strain *wdpks1*Δ-*1 *CYE. Similar results were seen with CYE from *W. dermatitidis *strains 8656 and *wdpks1*Δ-*1*.

The 2,2'-Azino-bis{3-ethylbenzothiazoline-6-sulfonic acid} [ABTS] assay results demonstrated the presence of phenoloxidase-like activity at the cell surface of the three *Wangiella *strains. Strain 8656 and the gene deleted strain produced similar results at an OD 420 nm, 0.131 and 0.133, whereas the complemented *wdpks1*Δ*-1*-501 strain revealed a lower activity, 0.087. The OD 420 nm determinations for the positive controls were 1.793 and 0.261 for commercial laccase and *C. neoformans*, respectively. When cytoplasmic yeast extract [CYE] were used, the activity was significantly higher with measurements of 0.527, 0.722, and 0.205 for strains 8656, *wdpks1*Δ*-1*, and *wdpks1*Δ*-1*-501, respectively. The experiments were repeated with similar results. Additional evidence for the presence of a phenoloxidase-like enzyme is demonstrated by the in situ polymerization of melanin in nondenaturing gels with CYE [Figure 7]. Similar results were observed with the commercially available *R. vernificera *laccase and the enzymatic activity of the CYE was abrogated with KCN.

### Mean inhibitory concentration

There were no significant differences in the mean inhibitory concentration [MIC] with the three strains for any of the drugs tested with or without visible melanization of the yeast cells in culture. The MIC determinations for the three strains were 1 μg/ml for amphotericin B, 0.0625 μg/ml for voriconazole, and 0.5 μg/ml for caspofungin. For *W. dermatitidis *strain 8656 the MIC with fluconazole was 0.5 μg/ml, whereas the MIC was 1 μg/ml for *wdpks1*Δ-*1 *and *pks1*Δ-*1*-501. The MICs were within values previously reported for strains of this fungus [29-32]. The MICs were performed three times with similar results.

### Killing assays

**Figure 8:**
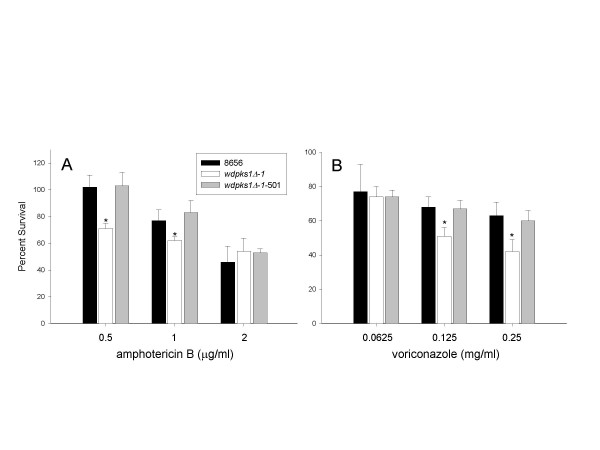
Antifungal drug time-kill assay. The rates of survival of strains 8656, *wdpks1*Δ-*1*, and *wdpks1*Δ-*1*-501 yeast cells grown for 7 days in YPD medium at 37°C after exposure to various concentrations of amphotericin B [A] or voriconazole [B] for 2 h compared to those of fungal cells incubated in PBS. Values are averages and brackets represent standard errors of the means for three measurements. *P *values were calculated by comparing wild-type and *wdpks1*Δ-*1 *cells using the Student's *t *test. *, *p *< 0.01. Similar results were obtained in three separate experiments.

**Figure 9:**
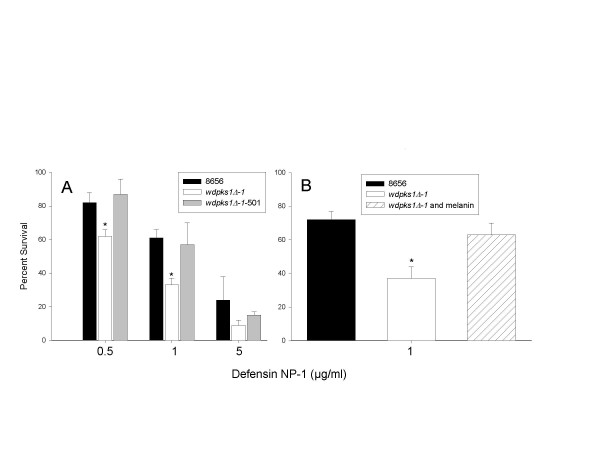
Defensin NP-1 time-kill assay. A] The rates of survival of strains 8656, *wdpks1*Δ-*1*, and *wdpks1*Δ-*1*-501 yeast cells grown for 7 days in YPD medium at 37°C after exposure to various concentrations of NP-1 for 30 min compared to those of fungal cells incubated in PBS. B] Survival of strain 8656 and *wdpks1*Δ-*1 *after exposure to 1 μg/ml of NP-1 and of *wdpks1*Δ-*1 *after exposure to 1 μg/ml NP-1 incubated with melanin particles derived from the gene deleted strain for 1 h prior to the use of the defensin solution in the assay. *P *value was calculated by comparing *wdpks1*Δ-*1 *cells treated with NP-1 with or without pre-incubation with melanin particles using the Student's *t *test. Values are averages of the standard errors of the means for three measurements. *, *p *< 0.01.

Cells from the darkly pigmented strains 8656 and *wdpks1*Δ*-1*-501 were significantly less susceptible to amphotericin B than the *wdpks1*Δ*-1 *strain [Figure 8A]. The differences were statistically significant for concentrations of 0.5 and 1 μg/ml, respectively. For voriconazole, the melanin deficient strain was more susceptible at higher [0.125 and 0.25 μg/ml] concentrations of the drug [Figure 8B]. The *wdpks1*Δ*-1 *strain was also more susceptible to NP-1 defensin at concentrations of 0.5 and 1 μg/ml [Figure 9A]. The addition of melanin particles for 1 hour prior to the use of the peptide in the time kill assay significantly reduced the efficacy of killing of *wdpks1*Δ*-1 *yeast cells [figure 9B].

**Figure 10:**
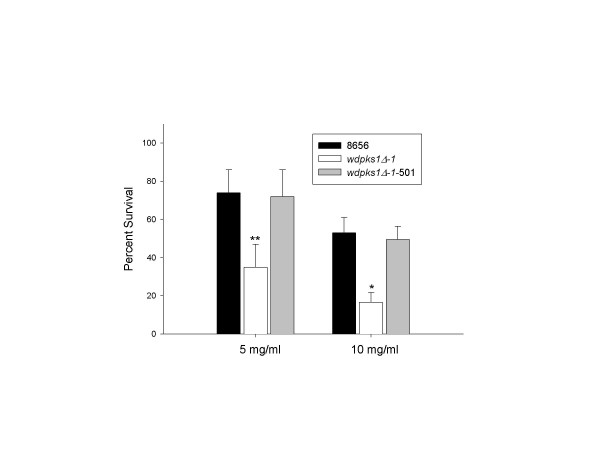
Survival of *W. dermatitidis *yeast cells in the presence of lysing enzymes. Strain 8656, *wdpks1*Δ-*1*, and *wdpks1*Δ-*1*-501 yeast cells grown for 7 days in YPD medium at 37°C and incubated in different concentration of Sigma lysing enzymes. Survival was compared to fungal cells incubated in PBS. The melanin-deficient mutant was significantly more susceptible to killing by incubation in lysing enzymes. Values are of the standard errors of the means for three measurements. *, *p *< 0.001 and **, *p *< 0.01. The experiment was repeated with similar results.

**Figure 11:**
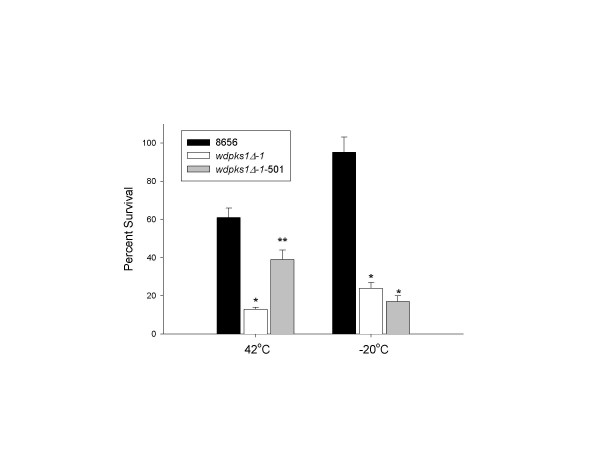
Susceptibility to heat and cold. Strain 8656, wdpks1Δ-*1*, and *wdpks1*Δ-*1*-501 yeast cells grown for 7 days in YPD medium at 37°C and incubated at 42°C for 2 h or -20°C overnight. Survival was compared to fungal cells incubated in PBS. Values are averages of the standard errors of the means for three measurements. *, *p *< 0.001 and **, *p *< 0.01. The experiment was repeated with similar results.

The lysing enzymes were significantly more toxic to the melanin-deficient mutant, having a reduction in survival of 50% or more than the wild-type or reconstituted strains [Figure 10]. The wild type and the reconstituted strains were not significantly different in their relative susceptibility. Exposure of *wdpks1*Δ*-1 *yeast cells to -20°C or 42°C significantly reduced their survival [Figure 11]. The advantage of the wild-type strain in surviving heat was significantly more pronounced than that measured for cold stress. Interestingly, the reconstituted strain *wdpks1*Δ*-1*-501 was more susceptible to both heat and cold compared to the wild-type strain. There was no difference between the survival of the melanin-deficient mutant and the reconstituted strain exposed to -20°C, *p *= 0.063. In contrast, significantly more *wdpks1*Δ*-1 *yeast cells were killed than *wdpks1*Δ*-1-*501 yeast cells at 42°C, *p *< 0.001.

## Discussion

Although melanin production occurs in diverse organisms, the mechanisms for melanization at specific locations, such as in the cell wall of fungi, are poorly understood. The regulation of melanin has proven complex in certain fungi, such as in *C. neoformans *where melanin formation can be catalyzed by at least 2 distinct laccases [33,34] and pathways like the cAMP cascade [33,35] and metal transporters [36] influence melanin synthesis. In *W. dermatitidis*, disruption of the polyketide synthase gene *WdPKS1 *results in an melanin-deficient phenotype [2]. However, transforming melanin-deficient strains generated by UV mutagenesis with *WdPKS1 *fails to restore melanin production in all mutants [2], indicating that there are additional genes or regulatory pathways involved in melanin production in *W. dermatitidis*. In fact, we have recently described the potential role for a hexapeptide in the synthesis of DHN melanin in *W. dermatitidis *[37], which suggests that the production of melanin in this pathogenic organism is more complicated than previously thought.

Disruption of *WdPKS1 *in *W. dermatitidis *results in a decrease in virulence in mice and cells deficient in the gene are more susceptible to killing by neutrophils [2]. Our current study begins to explore potential reasons for these effects. The cell wall of the late stationary phase *wdpks1*Δ*-1 *yeast is nearly 40% thinner than those of the wild type 8656 and the complemented *wdpks1*Δ*-1*-501 strain cultured identically [Figure 3]. Additionally, the melanin isolated from the mutant strain cultured for 10 days in YPD comprised only 4.6% of the dry yeast cell mass compared to 16.1 and 15.1% for the pigmented wild-type and reconstituted strains, respectively. The values for the strains producing cell wall melanin is higher than previously reported [12], however in the prior work the analysis was an estimation by a spectrophotometric method and used 36 to 48-h-old cultures [late log phase]. For comparison, melanin makes up 15.7% of the weight of pigmented *C. neoformans *cells cultured for 10 days [38]. The cell wall measurement for the wild-type strain is in accordance with prior determinations from transmission electron microscopy studies on *W. dermatitidis *[39]. The appearance of the cell wall of the mutant *wdpks1*Δ*-1 *is similar to a melanin-deficient mutant [3,40] and to thin-walled less-melanized yeast from 1- to 3- day-old cultures [41,42].

A reduction in the thickness of the cell wall and the absence of a polymerized melanin layer results in an increased susceptibility to killing by amphotericin B, voriconazole and defensin NP-1 [Figures 7 and 8]. Previous studies have shown that MIC is not a sensitive measurement of the ability of melanization to affect susceptibility to antifungal agents and that finding was confirmed here for *W. dermatitidis *[43]. Nevertheless, killing assays revealed that cells expressing cell wall associated melanin where significantly less susceptible to antifungal agents. The discrepancy between MICs and killing-assays could result from the fact that young daughter cells produced in exponentially growing *W. dermatitidis *cultures have less melanin than older mother cells or stationary phase cells [41,42], which results in the daughter cells having a greater susceptibility to the antifungal drugs. It has been shown that *C. neoformans *buds must synthesize melanin de novo [44] and transmission electron micrographs suggest that this is also true for *W. dermatitidis *[41,45]. Hence, to determine the effect of antifungals on *W. dermatitidis *cells with and without melanin is best determined by killing assays.

The melanin-deficient *wdpks1*Δ*-1 *strain was significantly more susceptible to killing by exposure to lysing enzymes and to heat or cold than were the wild-type strain and the complemented strain [Figure 10 and 11]. Also, the melanin-deficient cells were more susceptible to damage by rapid freezing in liquid nitrogen [Figure 5]. These results were not particularly surprising, because we have previously shown that inhibition of melanization of *W. dermatitidis *yeast by culture with tricyclazole significantly increases killing by zymolyase or glusulase lysis compared to melanized cells [12]. Most likely the melanin binds and inactivates certain hydrolytic enzymes or combines with substrates to protect cells from the enzymatic degradation of their cell walls leading to death by lysis [reviewed in [46]]. Similarly, we have also shown that melanized *C. neoformans *have significantly greater survival during exposure to extremes in temperature than cells lacking melanin [47]. Thus, the reduced susceptibility of wild-type cells of *W. dermatitidis *to heat probably similarly reflects the capacity for melanin to absorb heat energy, dissipate it, and temporarily shield against heat damage [reviewed in [5]]. Melanin in the cell wall may also protect against freezing temperatures by providing mechanical stability to the cell against shearing stress produced by ice crystals. In some fungal pathogens of plants, melanin is essential for the ability of the fungal cells to resist high internal pressures [reviewed in [14]]. Interestingly, invasive growth in *W. dermatitidis *is dependent upon melanin biosynthesis [48]. While it is not clear why the reconstituted *wdpks1*Δ*-1-*501 strain was more susceptible than the wild type to injury from heat and cold, we speculate that the insertion of 13 kb of extra genetic information during complementation may have resulted in a positional effect on downstream genes or in a chromosomal rearrangement that increased sensitivity to temperature shifts [2].

The residues isolated from the wild type and complemented strains after treatment with enzymes, denaturant and hot acid were similar in size and morphology to the cells from which they were derived, whereas the nondescript particles from the *wdpks1*Δ*-1 *yeast were notably smaller [Figure 3]. Nonetheless the melanin-binding antibody reacted with the residues from all three types of cells [Figure 4], indicating that even the small particles from *wdpks1*Δ*-1 *were comprised of a melanin-like material. When cell wall integrity was perturbed by liquid nitrogen and then incubated with the melanin-binding antibody, melanin-like compounds were only detected within the cytoplasm of the melanin-deficient mutant, whereas cells of the wild type and reconstituted strains similarly treated were labeled at the cell wall and in the cytoplasm [Figure 5]. Support for our contention that the small debris and the cell-sized particles were melanin-like is provided by the ESR analysis that revealed the presence of stable free radicals in the material isolated from the three *W. dermatitidis *strains [Figure 6]. ESR spectroscopy has been used previously to study and define melanins based on the properties of unpaired electrons present in melanin [22] and it has also been used to identify pigments as melanins in several fungi, including *C. neoformans *[38] and *P. brasiliensis *[24].

We hypothesized that if *W. dermatitidis *produces different types of melanin and because all melanins, except for DHN-melanin, contain nitrogen in their chemical structure, then the presence of nitrogen in the samples can serve as a proof that at least one other type of melanin is present in the samples. Elemental analysis demonstrated considerable percentage of nitrogen in all samples. We found that the wild type 8656 and the complemented *wdpks1*Δ*-1*-501 strains had similar C:N ratios [18.6C:1N and 19N:1C, respectively]. While this ratio was higher than that previously reported for *A. niger *melanin, 14.5C:1N [49] and 16C:1N [50], it was significantly lower than the C:N ratio in the melanin from the mutant *wdpks1*Δ*-1 *yeast [26.2C:1N]. Our previous studies on the elemental analysis of DOPA-derived melanins have shown ratios of 9C:1N for synthetic melanin and 8C:1N for melanin from *C. neoformans *and *S. officinalis *[49]. Some of the variations can be attributed to the heterogeneity in polymerization of intermediate structures of the melanogenic pathway that results in the formation of a mixture of various polymerized structures. This is particularly noteworthy for the melanins generated by the wild type and reconstituted strains, since they are comprised of melanin generated via *WdPKS*-dependent [cell wall] and independent [cytoplasmic] pathways.

Our data strongly supports the conclusion that the small particles isolated from the melanin-deficient *wdpks1*Δ*-1 *strain are melanin-like compounds. One possibility is that precursors for melanin exist within the fungal cells and that they are polymerized during the isolation procedure. However, we determined that the CYE of the strains of *W. dermatitidis *used in our studies had phenoloxidase activity capable of catalyzing the formation of melanin from a phenolic precursor. We also show that strain *wdpks1*Δ-*1 *is capable of melanization in the presence of L-DOPA [Figure 1]. The final step of DHN synthesis is purported to be an oxidation reaction, which could be accomplished by a phenoloxidase. In fact, based on biochemical studies n the 1980s, it has been suggested that a laccase was responsible for the polymerization and oxidation of DHN into melanin [6]. Interestingly, phenoloxidase activity has been demonstrated in *A. fumigatus *[51] and genes homologous to laccases have been indentified in *A. fumigatus *[52] and *A. nidulans *[53]. Our results suggest that a phenoloxidase that may be involved in DHN melanin synthesis can additionally produce *WdPKS*-independent melanin in *W. dermatitidis*.

There are numerous electron dense granules within the cytoplasm of *W. dermatitidis *cells [Figure 2] and it is possible that these granules may be polymerized melanin precursors or shunt metabolites. These granules could then coalesce into larger spheres during the melanin isolation process or become compartmentalized within vacuoles in the cytoplasm. Several studies of *W. dermatitidis *by transmission electron microscopy have revealed the presence of larger "dense bodies" that are electron opaque vacuoles typically less than 0.5 μm in diameter [39,41,42,45,54,55]. The micrographs obtained during our study with 7-day-old cells do not have well represented vacuoles. However, the visualization of these structures varies greatly with the method of preparation [55] and the age of the cell [41,42,45]. It is noteworthy that deposition of melanin in electron dense intracellular organelles ["melanosomes"] has been demonstrated in *Fonsecaea pedrosoi *[56,57] and similar structures have also been reported in *Cladosporium carrionii *and *Hormoconis resinae *[58]. Abolition of DHN melanin formation via the polyketide synthase pathway in *F. pedrosoi *has recently allowed for the identification of a second type of melanin in this fungus [59]. Melanin production by the addition of phenolic substrate after abolition of the polyketide synthase pathway has been demonstrated in other fungi, such as *Thielaviopsis basicola *and *Verticillium dahliae*, resulting in pigment formation that differs from that produced in the absence of blocking the polyketide synthase [60,61]. Furthermore, although visually albino, spherical melanin-like particles have recently been isolated from *C. albicans *yeast [62]. These findings are consistent with the identification of small melanin-like particles in *W. dermatitidis wdpks1*Δ*-1 *yeast and suggest that the gene defect halts the polymerization of melanin in the fungal cell wall but not the synthesis of melanin within the cytoplasm or vacuoles of the cells. Consistent with this is the finding that the activity of the phenoloxidase-like enzyme is substantially greater in the cytoplasm of *W. dermatitidis *compared to the cell surface. The low level of the enzymatic activity on the cell surface may account for the light brown coloration achieved in the *wdpks1*Δ-*1 *cells grown in YPD, which contains small amounts of phenolic compounds. The light brown cells do not produce enough melanin to form polymerized shells in their walls, since cell shape is not maintained in the isolation of melanin from the melanin-deficient yeast. When grown in the presence of L-DOPA, the gene deficient yeast cultures are visibly black and particles derived from them by the melanin isolation procedure maintain the size and shape of the yeast, though there is more pigment produced within the cell than at the cell surface. Possibly when the genome of this pathogen becomes deciphered, the precise nature of the additional pathways for the synthesis of melanin will become evident.

In summary, we have shown that *W. dermatitidis *produces more than one type of melanin. We have also documented that the deposition of the dominant *WdPKS*-dependent DHN melanin polymer in the cell significantly improved the pathogen's capacity to resist diverse stressors. Although the role of intracellular melanin-like compound(s) remains uncertain, our results suggest that they continue to be an area ripe for future investigations.

## Materials and methods

### Fungal strains and media

The laboratory wild-type strain of *W. dermatitidis *8656{ATCC 34100 [*Exophiala dermatitidis *CBS 525.76]}, a strain with a disrupted polyketide synthase gene [*wdpks1*Δ*-1*], and its complemented strain [*wdpks1*Δ*-1*-501] have been described [2]. Routine propagation of these strains was in YPD [2% peptone, 1% Bacto Yeast extract, and 2% dextrose] at 37°C with shaking at 150 rpm. The mutant was also grown in a defined chemical medium [minimal media: 15.0 mM glucose, 10.0 mM MgSO_4_, 29.4 mM KH_2_PO_4_, 13.0 mM glycine, and 3.0 μM thiamin; pH 5.5] with or without 1 mM L-3,4-dihyroxyphenylalanine [L-DOPA, Sigma Chemical Co.; St. Louis, MO]. The minimal medium cultures where incubated in the dark to minimize potential a L-DOPA autopolymerization.

### Transmission electron microscopy

Yeast cells were grown in YPD medium for 7 days, washed in phosphate-buffered saline [PBS; 0.137 M NaCl, 0.003 M sodium phosphate; H 7.4], and fixed overnight at room temperature with 2.5% glutaraldehyde in 0.1 M cacodylate. Next, the cells were incubated overnight in 4% formaldehyde-1% glutaraldehyde-0.1% PBS and then in 2% osmium for 1.5 h. Dehydration was accomplished by serial washing in graded ethanol solutions of 50 to 95% for 10 min, followed by two final washes in 100% ethanol for 15 min. Cells were embedded in Spurr's resin, sectioned onto grids, and subjected to transmission electron microscopy with a model 102 electron microscope [Siemens, Berlin, Germany] to obtain electron micrographs.

### Isolation of melanin particles

Melanin particles were isolated from yeast grown for 10 d by a modification of a described methodology [49]. Briefly, cells were collected by centrifugation, autoclaved, washed with PBS, air dried, and the pellets were weighed. The cells were also suspended in 1.0 M sorbitol-0.1 M sodium citrate [pH 5.5], after which cell wall lysing enzymes [from *Trichoderma harzianum*; Sigma, Cleveland, OH] were added at 10 mg/ml and the suspension was incubated at 30°C overnight. The resulting protoplasts were collected by centrifugation, washed with PBS, and treated with 1.0 mg/ml proteinase K [Roche Laboratories; Indianapolis, IN] in a reaction buffer [10.0 mM Tris, 1.0 mM CaCl_2_, and 0.5% SDS; pH 7.8] at 37°C overnight. The debris was then collected, washed with PBS, and boiled in 6.0 M HCl for 1 h. After collecting the remaining particles by centrifugation, they were washed again in PBS and then dialyzed extensively against distilled water, air dried and weighed. This procedure has been shown to solubilize non-melanized *C. neoformans*, *Paracoccidioides brasiliensis*, *Saccharomyces cerevisiae*, and *Candida albicans *yeast cells [24,49]. Chitin is also solubilized by this treatment [24].

### Scanning electron microscopy

The particles collected after treatment of yeast cells with enzymes, denaturant and hot acid were incubated in 2.5% glutaraldehyde for 1 h at room temperature. The samples were then applied to a poly-L-lysine-coated coverslip and serially dehydrated in alcohol. The samples were fixed in a critical-point drier [Samdri-790; Tousimis, Rockville, Md.], coated with gold-palladium [Desk-1; Denton Vacuum, Inc., Cherry Hill, N.J.], and viewed with a JEOL [Tokyo, Japan] JSM-6400 scanning electron microscope.

### Immunofluorescence microscopy

Slides were prepared with 10 μl suspensions containing 10^6 ^particles derived from yeast cells subjected to enzymes, denaturant and hot acid. The samples were placed on poly-L-lysine-coated slides and air-dried. The slides were thoroughly blocked with Superblock^® ^[Pierce, Rockford, IL] blocking buffer for 1 h at 37°C followed by incubation with 10 μg/ml of the melanin-binding monoclonal antibody [mAb] 6D2 for 2 h at 37°C. MAb 6D2 [μκ] was generated against melanin derived from *C. neoformans *and is known also to specifically bind other types of melanins, including fungal melanin from *Sporothrix schenckii *[26], *Scytalidium dimidiatum *[28], *Paracoccidioides brasiliensis *[24], *Pneumocystis spp*. [27], *H. capsulatum *[25], *B. dermatitidis *[20], synthetic tyrosine derived melanin [49], melanin from the cuttlefish *Sepia officinalis *[49], and human melanin [63]. The antibody does not bind to amelanotic fungal cells [49,64]. After washing, the slides were incubated with a 1:100 dilution of fluorescein isothiocyanate [FITC] -conjugated goat anti-mouse [GAM] IgM [Southern Biotechnologies Associates, Inc; Birmingham, AL] for 1.5 h at 37°C. The slides were washed, mounted using a 50% glycerol, 50% PBS, and 0.1 M *N*-propyl gallate solution, and viewed with an Olympus AX70 microscope [Melville, NY] equipped with a fluorescent filters. Negative controls consisted of slides incubated with mAb 5C11 [μκ], which binds mycobacterial lipoarabinomannan [65], as the primary Ab or FITC-labeled Ab alone.

Additionally, cells grown for 15 days were placed in liquid nitrogen for 10 min to disrupt the integrity of the cells and aliquots of the suspensions were dried on slides. The slides were prepared for immunofluorescence microscopy as described above except tetramethyl rhodamine isothiocyanate [TRITC]-GAM IgM [Southern Biotech.] was used in place of FITC.

### Electron spin resonance spectroscopy

Electron spin resonance [ESR] spectroscopy was performed on particles isolated from yeast cells grown in YPD media as described [23], except that a Gunn diode was used as the microwave source.

### Elemental analysis

Carbon, nitrogen, and oxygen analyses on lyophilized melanin samples were performed by Quantitative Technologies Inc. [Whitehouse, NJ]. Briefly, melanin samples were converted into gases such as CO_2_, H_2_O, and N_2 _by combustion. The product gases were separated under steady-state conditions and the percentage of each element in the samples was measured as a function of thermal conductivity. Ratios of C:N were calculated by dividing the percentage of each element in the samples by their respective atomic weights.

### Polyacrylamide gel electrophoresis [PAGE] analysis for phenoloxidase-like activity

The phenoloxidase-like activity of cytoplasmic yeast extract [CYE] for catalyzing the polymerization of melanin from L-DOPA was determined as described previously [23]. Briefly, *W. dermatitidis *yeast cells were collected, suspended in 0.1 M Na_2_HPO_4 _with protease inhibitor, and treated for 6 min in a bead beater at 2-min intervals alternating with 5 min on ice. Supernatants were separated from cellular debris by centrifugation at 8,000 rpm for 10 min. Supernatants and commercial laccase were separated by 10% PAGE electrophoresis run at 18 mA overnight under nondenaturing conditions. As controls, samples were treated with 0.1 M KCN, an irreversible inhibitor of phenoloxidase activity, before being loaded onto the gel. Gels were incubated with 1 mM L-DOPA in 0.1 M citric acid-0.2 M Na_2_HPO_4 _[pH 6.0] buffer for 6 h.

### Quantitative phenoloxidase assay

Yeast cells were grown in asparagine medium [1 gm asparagine, 0.1 gm of MgSO_4_, 3 gm of Na_2_HPO_4_, CaCl2 per liter, pH 7.0] with glucose [1.5 gm/l] for 72 h at 37°C. The cells were collected by centrifugation, washed with Na_2_HPO_4_, and transferred into asparagine medium without glucose for 36 h at 37°C. The strains were collected by centrifugation, washed with Na_2_HPO_4_, and diluted to 1 × 108 cells/ml in Na_2_HPO_4_. A final concentration of 1 mM 2,2'-Azino-bis{3-ethylbenzothiazoline-6-sulfonic acid} [ABTS; Sigma] was achieved by adding 100 μl of 10 mM ABTS to 900 μl of a yeast cell suspension. After incubation at 30°C for 2 h, the cells were removed after centrifugation and the absorbance readings of the solutions were measured at 420 nm. A yeast cell suspension without ABTS was used as a baseline. Commercially produced laccase from *Rhus vernificera *[activity, 50 U per mg of solid; Sigma] was used as a positive control at 1 unit in 1 mL Na_2_HPO_4_. Additionally, since *C. neoformans *produces laccase that is expressed on its cell surface [66], *C. neoformans *strain 24067 from the American Tissue Culture Collection [Rockville, MD] was cultured and assayed as above. The experiments were repeated using CYE from similar numbers of cells as those used for the whole cell assay.

### MIC determination

MICs were determined by the standardized protocol for yeasts developed by the National Committee for Clinical Laboratory Standards [NCCLS, M27A] [67]. Voriconazole and fluconazole were provided by Pfizer [Sandwich, England], caspofungin was purchased from Merck [Whitehouse Station, NJ], and amphotericin B was purchased from Gibco [Invitrogen Corp., Carlsbad, CA]. Briefly, yeast cells grown for 5 days were suspended in sterile normal saline and diluted to a concentration of 2 × 10^6 ^cells/ml. Cell counts were determined by hemacytometer. The suspensions were diluted 1:1000 in RPMI 1640 medium with L-glutamine, without bicarbonate, and buffered to pH 7.0 with 0.165 M morpholinopropanesulfonic acid [MOPS]. Polystyrene tubes containing 0.1 ml aliquots of an antifungal at 10 times the final drug concentration were inoculated with 0.9 ml of the diluted suspensions. Final drug concentrations ranged from 0.063 to 4 μg/ml for amphotericin B, 0.125 to 64 μg/ml for caspofungin, and from 0.007 to 16 μg/ml for voriconazole and fluconazole. The MIC's were recorded after incubation at 35°C for 72 hours. MIC's for voriconazole, amphotericin B and caspofungin were defined as the lowest concentration at which there was an absence of growth. The MIC for fluconazole was the lowest drug concentration that achieved 80% growth inhibition compared to the drug-free control.

### Time-kill assays

Yeast cells grown for 7 days were suspended in sterile normal saline at a density of 2.2 × 10^3 ^cells/ml. Cell counts were determined by hemacytometer. Microcentrifuge tubes containing 0.1 ml aliquots of an antifungal at 10 times the final concentration were inoculated with 0.9 ml of the yeast suspensions. Final drug concentrations ranged from 0.0625 to 0.25 μg/ml for voriconazole and 0.5 to 2 μg/ml for amphotericin B. After incubation at 35°C for 2 hours, aliquots were plated on YPD agar to determine their viabilities as measured by CFU. Survival was compared to that of fungal cells incubated in PBS.

The defensin NP-1 [gift of R. Lehrer, Los Angeles, CA] was also used in time-kill studies. NP-1 is a defensin derived from the neutrophils of rabbits with the sequence of VVCACR-RALCLPRERRAGFCRIRGRIHPLCCRR-NH_2_. Final concentrations of peptide tested ranged from 0.5 to 10 μg/ml and the exposure period was shortened to 30 min. To evaluate whether the presence of melanin could influence the outcome of the interaction of the defensin with *W. dermatitidis*, the gene-deleted strain was also exposed to a solution of NP-1 pre-incubated with 20 μg of melanin particles derived from strain *wdpks1*Δ*-1 *yeast cells.

### Enzymatic degradation assay

*W. dermatitidis *yeast grown for 7 days were washed in PBS and incubated for 2 h at room temperature with 5 or 10 mg/ml of lysing enzyme [from *Trichoderma harzianum*; Sigma Chemical Corp., Cleveland, OH], which contains protease, cellulose and chitinase activites. The cells were washed twice in PBS and then plated for CFU. The survival was determined relative to CFUs obtained with cells incubated in PBS alone.

### Heat and cold exposure

After 7 days of growth, yeast cells were washed in PBS and either incubated in a 42°C water bath for 1 h or frozen at -20°C for 24 hours. The frozen cells were thawed at room temperature. Aliquots were plated onto YPD agar plates subsequently incubated at 37°C. Survival rates were determined by counting the number of colonies relative to those of non-exposed cells [control]. Control cells were plated at the same time the experimental cells were heated or frozen to avoid changes in cell number.

### Statistics

Statistical significance was determined using the Student's t-test [Primer; McGraw-Hill, New York, New York, USA].

## Abreviations

DNH: dihydroxynaphthalene

PBS: phosphate-buffered saline

mAb: monoclonal antibody

FITC: fluorescein isothiocyanate

GAM: goat anti-mouse

TRITC: tetramethyl rhodamine isothiocyanate

ESR: electron spin resonance

ABTS: 2,2'-Azino-bis{3-ethylbenzothiazoline-6-sulfonic acid}

CYE: cytoplasmic yeast extract

MIC: mean inhibitory concentration

MOPS: morpholinopropanesulfonic acid

CFU: colony forming unit

L-DOPA: L-3,4-dihyroxyphenylalanine

OD: optical density

## Authors' contributions

Paolo, William F. Jr: isolated melanin, performed MIC and killing assays.

Dadachova, Ekaterina: design and analysis of characterization of melanin data.

Mandal, Piyali: performed lysing and theromotolerance assays.

Casadevall, Arturo: design and analysis of experimental data, critical editing.

Szaniszlo, Paul J.: produced mutant strains; design and analysis of experimental data, critical editing.

Nosanchuk, Joshua D.: oversaw all aspects of study- performed electron microscopy, microbicidal peptide assay, ABTS assay, and laccase gels; wrote the manuscript.
